# Local anesthetic lidocaine-inducible gene, growth differentiation factor-15 suppresses the growth of cancer cell lines

**DOI:** 10.1038/s41598-022-18572-3

**Published:** 2022-08-25

**Authors:** Keiko Haraguchi-Suzuki, Reika Kawabata-Iwakawa, Toru Suzuki, Takashi Suto, Tomonori Takazawa, Shigeru Saito

**Affiliations:** 1 Intensive Care Unit, Gunma University Hospital, 3-39-15, Showa-machi, Maebashi, Gunma 371-8511 Japan; 2 Division of Integrated Oncology Research, Initiative for Advanced Research, Gunma University, 3-39-15, Showa-machi, Maebashi, Gunma 371-8511 Japan; 3 Laboratory for Immunogenetics, RIKEN Center for Integrative Medical Sciences, 1-7-22, Suehiro-cho, Tsurumi-ku, Yokohama, Kanagawa 230-0045 Japan; 4 Department of Anesthesiology, Gunma University Hospital, 3-39-15, Showa-machi, Maebashi, Gunma 371-8511 Japan

**Keywords:** Biochemistry, Cancer, Drug discovery, Molecular biology, Oncology

## Abstract

Administration of local anesthetics, such as lidocaine, in the perioperative period improves outcomes of cancer patients. However, its precise mechanism is still unresolved. The growth of human cancer cell lines, including HeLa cells, are suppressed by lidocaine treatment. We identified that growth differentiation factor-15 (GDF-15) was commonly upregulated in lidocaine-treated cancer cell lines. GDF-15 is a divergent member of the transforming growth factor-β (TGF-β) superfamily and it is produced as an unprocessed pro-protein form and then cleaved to generate a mature form. In lidocaine-treated HeLa cells, increased production of GDF-15 in the endoplasmic reticulum (ER) was observed and unprocessed pro-protein form of GDF-15 was secreted extracellularly. Further, lidocaine induced apoptosis and apoptosis-inducible Tribbles homologue 3 (TRIB3) was also commonly upregulated in lidocaine-treated cancer cell lines. In addition, transcription factor C/EBP homologous protein (CHOP), which is a positive regulator of not only GDF-15 but TRIB3 was also induced by lidocaine. Lidocaine-induced growth suppression and apoptosis was suppressed by knockdown of GDF-15 or TRIB3 expression by small interference RNA (siRNA). These observations suggest that lidocaine suppresses the growth of cancer cells through increasing GDF-15 and TRIB3 expression, suggesting its potential application as cancer therapy.

## Introduction

Increase in longevity in humans is associated with an increase in the number of cancer patients^1^. Cancer patients require surgery and anesthesia, along with analgesia for pain control. Both opioids and local anesthetics are commonly administered for analgesia. However, the type of analgesic administered influences prognosis^2^. Previous studies demonstrated that administration of local anesthetics decreased the incidence of recurrence or metastasis^3,4^. Local anesthetics inhibit both sympathetic system activation and immunosuppression caused by surgery through blocking the neuroendocrine stress axis, indicating that local anesthetics are beneficial in cancer patients^5,6^. In addition, unlike ester-linked types, amide-linked local anesthetics, such as lidocaine, have direct anti-tumor effects on cancer immunity, metastasis and apoptosis^7–10^. Lidocaine was first synthesized in the mid-nineteenth century and is commonly used in clinical practice for pain relief and as an antiarrhythmic drug^11^. Local anesthetics, including lidocaine, act on voltage-gated sodium channels (VGSC). Although several cancer cells such as colon cancer SW620 cells express VGSC, it is controversial whether the anti-tumor effects of lidocaine are mediated via VGSC^10,12^. Therefore, the precise mechanisms of local anesthetic-related anti-tumor effects are still unresolved.

Growth differentiation factor-15 (GDF-15) belongs to the transforming growth factor-β (TGF-β) superfamily. GDF-15 plays diverse roles under physiological and pathological conditions in relation to inflammation, metabolism and cancer. Like other TGF-β superfamily proteins, GDF-15 is produced in a pro-protein form and undergoes disulfide linkage in the endoplasmic reticulum (ER) before cleavage of the N-terminal pro-peptide region. Mature GDF-15 is secreted extracellularly, together with the pro-peptide and pro-protein forms^13–15^. GDF-15 is also called as nonsteroidal anti-inflammatory drug (NSAID) activated gene-1 (NAG-1), because it was identified as an NSAID-induced gene based on the evidence that the development of colon cancer was prevented by the administration of NSAIDs^16,17^. Studies using transgenic mice expressing GDF-15 showed that GDF-15 exerts both tumor suppressive and progressive activity, suggesting that it has opposite roles during different stages of cancer^18–20^.

Tribbles pseudokinase is evolutionally conserved from Drosophila to mammalian. In Drosophila, Tribbles is critical for development through negatively regulating cell cycle regulator string/CDC25^21^. In human, one of the homologs of Drosophila Tribbles, Tribbles homologue 3 (TRIB3) is induced by cellular stress such as hypoxia and nutrient starvation^22,23^. In cancer, TRIB3 increases stemness of cancer cells through activating transcription factors which induce expression of stemness-related genes. In breast cancer, TRIB3 associates with Akt to inhibit degradation of transcription factor forkhead box protein O1 (FOXO1)^24^. In colorectal cancer, TRIB3 activates Wnt signaling pathway through interacting with transcription factors such as β-catenin and T cell factor 4 (TCF4)^25^. On the other hand, increased TRIB3 expression predicts a preferable prognosis in breast cancer^26^. In addition, TRIB3 induces apoptosis in ER stress-induced HeLa cells and lung cancer cell line A549 cells^27,28^.

C/EBP homologous protein (CHOP) is a family of leucine zipper transcription factor and is induced by cellular stress such as hypoxia, amino acid starvation and ER stress^29–31^. Importantly, GDF-15 promoter is activated by CHOP and activating transcription factor 3 (ATF3) in NSAIDs sulindac sulfate-treated colorectal cancer cell lines such as HCT116^32^. Besides, combination of CHOP and activating transcription factor 4 (ATF4) induces apoptosis through the induction of TRIB3 in ER stress-induced HepG2 cells^27^. These indicates that CHOP-induced GDF-15 and TRIB3 have significant roles in cancer.

In this study, we observed growth suppression of cancer cell lines, including cervical cancer HeLa, osteosarcoma U2OS, hepatocellular carcinoma HepG2 and colon cancer SW480 cells, by lidocaine treatment. Using RNA sequence analysis (RNA-seq), we identified changes in gene expression levels in lidocaine-treated cancer cell lines and GDF-15 was upregulated in a lidocaine concentration-dependent manner. Furthermore, apoptosis-inducible TRIB3 was also upregulated in these cells. Our findings might provide novel insights into the mechanism that lidocaine might be an effective adjunct to cancer therapy.

## Results

### Growth of cancer cell lines was suppressed by lidocaine treatment

**Figure 1:**
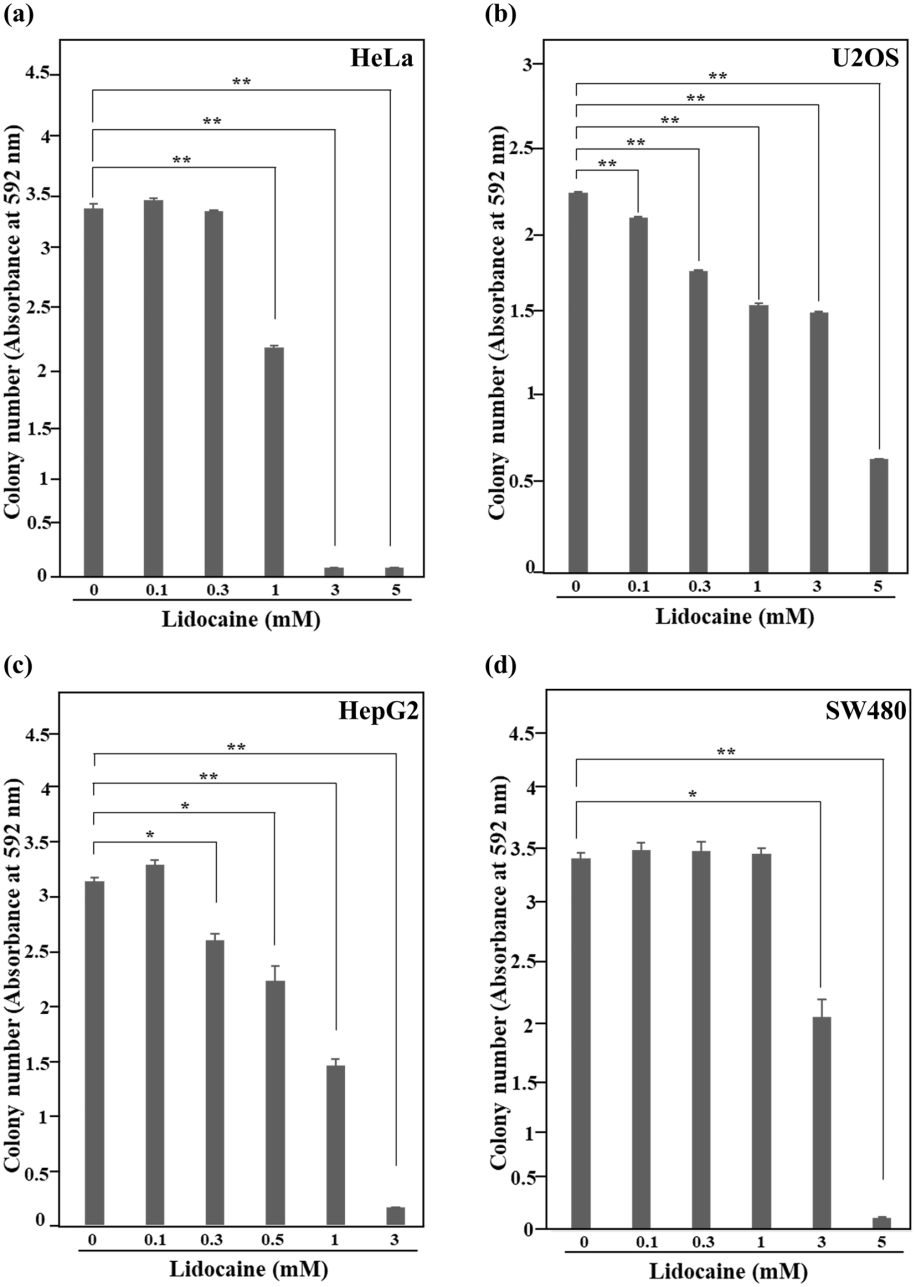
Growth suppression of cancer cell lines with lidocaine treatment. Colony formation assay. HeLa (**a**), U2OS (**b**), HepG2 (**c**) and SW480 (**d**) cells were treated with the indicated concentration of lidocaine and cultured for 7 days. The growth of each cell line was inhibited by lidocaine treatment in a concentration dependent manner. Each sample was assayed in triplicate and the results are presented as the mean ± SD. * *p* < 0.05, **p < 0.001.

To examine the effect of the amide-linked local anesthetic, lidocaine, on the growth of human cancer cell lines, colony formation assay was performed using cervical cancer HeLa, osteosarcoma U2OS, hepatocellular carcinoma HepG2 and colon cancer SW480 cells. Ropivacaine, which is also an amide-linked local anesthetic, was previously shown to inhibit the proliferation of HepG2 cells and doxorubicin-resistant U2OS cells^33,34^. It also suppressed metastasis of colon cancer SW620 cells^12^. Furthermore, combination therapy with ropivacaine and nutrient restriction suppressed the growth of HeLa cells^35^. Thus, we examined the effects of lidocaine on these and similar cell lines. The growth of HeLa cells was significantly suppressed by 1 mM lidocaine treatment. The growth of U2OS and HepG2 cells was suppressed by lower lidocaine concentrations of 0.1 mM and 0.3 mM, respectively, compared with that of HeLa cells. Conversely, the growth of SW480 cells was effectively suppressed by a higher lidocaine concentration of 3 mM. These results indicate that the growth of these cell lines was inhibited by lidocaine in a concentration-dependent manner, with the cell lines showing differential sensitivities to lidocaine-induced growth suppression (Fig. 1a–d).

### Comprehensive transcriptome sequence analysis of lidocaine-treated cancer cell lines

**Figure 2:**
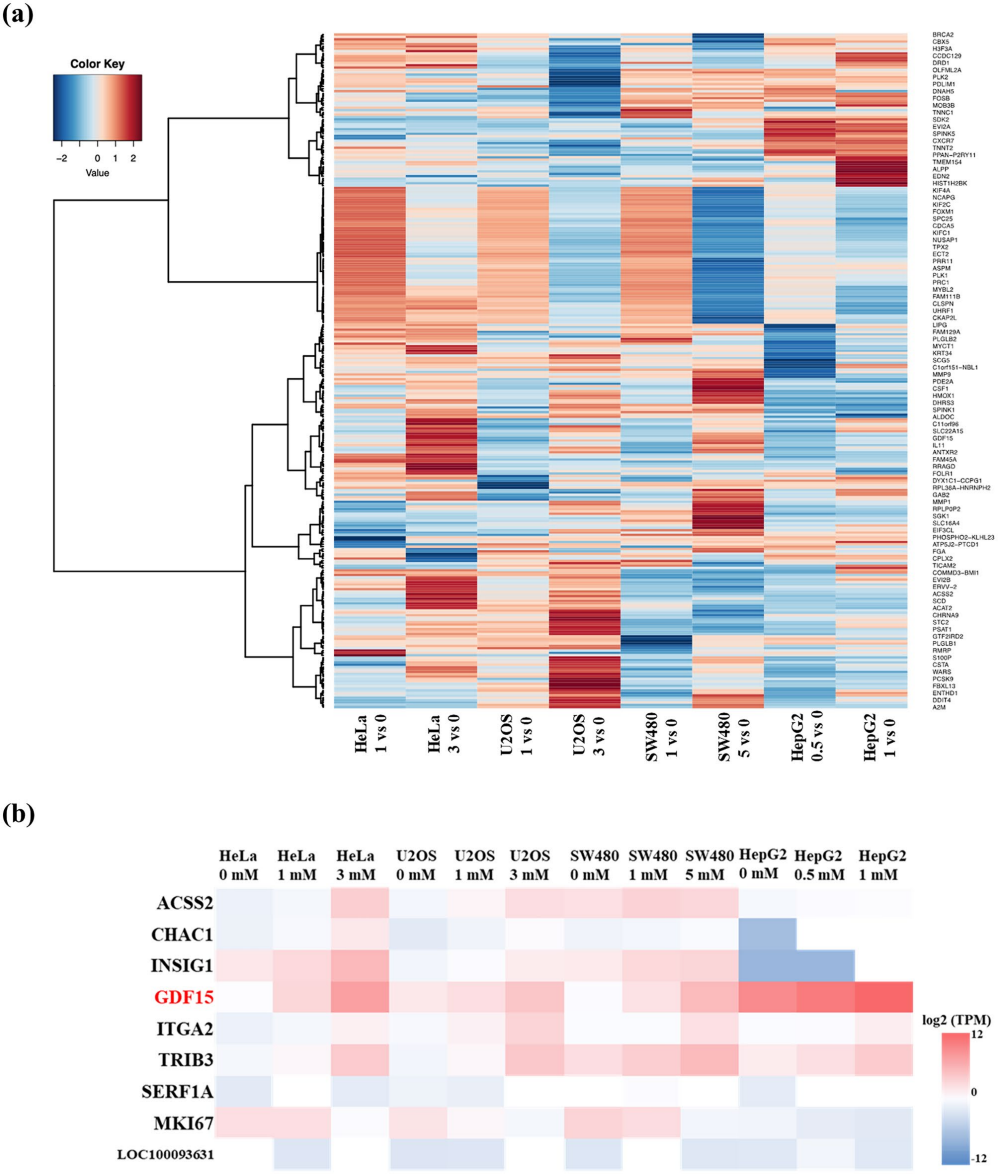
Transcriptome expression analysis of lidocaine-treated cancer cell lines. (**a**) Heatmap of z-scored log2 fold change values with adjusted *p* values of < 0.05, which were obtained from TCC-iDEGES-DESeq using the heatmap2 library on R package gplots_3.1.1 as follows: calculating z-scores, omitting “N/A” data, and hierarchical clustering with Ward’s method. Two hundred eighty-six genes had adjusted *p* values < 0.05 on at least one analysis. (**b**) Heatmap of commonly modified nine genes in examined cancer cell lines. The value represents the log2 fold change of transcripts per kilobase million (TPM).

To elucidate the mechanism of lidocaine-induced growth suppression of cancer cell lines, we examined the gene expression profile of the four cell lines with and without lidocaine treatment. HeLa, U2OS, SW480 and HepG2 cells were treated with the different concentrations of lidocaine, found to result in induction of significant growth inhibition (1 and 3 mM for HeLa and U2OS cells, 1 and 5 mM for SW480 cells and 0.5 and 1 mM for HepG2 cells). After treatment for 24 h, total RNAs were purified from the cells and high-quality RNA samples were used for RNA-seq using NextSeq500 (Illumina Inc.) as described in the Methods. Differentially expressed genes (DEGs) between two conditions were detected using TCC-iDEGES-DESeq pipeline on R (https://www.R-project.org/, version 4.0.3) package TCC (version 1.26.0). Genes with a false discovery rate (FDR, adjusted *p* value) of < 0.05 were defined as being significant DEGs. Two hundred eighty-six genes were extracted as DEGs on at least one analysis (Fig. 2a). In addition, we identified the commonly modified genes in the examined cancer cell lines, and found that the expression levels of nine genes were significantly changed in response to lidocaine (Fig. 2b and Table 1). Among these genes, GDF-15 was upregulated in a lidocaine dose-dependent manner, suggesting that GDF-15 plays a significant role in lidocaine-induced growth suppression of cancer cell lines.

**Table 1:** Commonly modified genes in cancer cell lines with lidocaine treatment.

	HeLa_l_vs_0		HeLa_3._vs_0		U2OS_l_vs_0		U2OE_3_vs_0		SW480_l_vs_0		SW480_5_vs_0		HepG2_0.5_vs_0		HepG2 _1_vs_0	
Gene symbol	m.value	q.value	m.value	q.value	m.value	q. value	m.value	q.value	m.value	q.value	m.value	q.value	m.value	q.value	m.value	q.value
*GDF15*	1.73	l.08E−16	4.15	6.49 E−10	0.46	1.000	1.49	0.468	1.25	1.06E−05	2.97	0.024	0.72	0.0016	1.48	0.088
*TRIB3*	1.04	3.94E−05	2.92	7.47E−05	1.24	0.001	3.30	2.17E−05	0.64	0.149	1.49	1.000	0.54	0.256	1.38	0.16
*ITGA2*	0.96	2.59E−05	2.11	0.018	0.99	0.0048	2.25	0.017	− 0.07	1.000	1.30	1.000	0.11	1.000	0.80	1.00
*ACSS2*	0.96	0.0028	3.64	1.32E−07	1.24	3.07 E−04	2.17	0.030	0.54	0.572	0.26	1.000	0.45	1.000	0.54	1.00
*INSIG1*	0.63	0.029	2.06	0.020	0.98	0.024	1.71	0.235	0.72	0.036	0.78	1.000	0.060	1.000	− 2.32	1.00
*CHAC1*	1.01	0.030	2.36	0.012	1.42	0.037	2.74	0.0061	0.37	1.000	0.90	1.000	− 6.58	1.000	− 2.32	1.00
*LOC100093631*	14.18	5.15E−20	1.71	1.000	0.15	1.000	− 14.30	8.99E−07	− 12.43	4.99E−18	0.011	1.000	2.00	9.44E−14	− 0.077	1.00
*SERF1A*	− 11.74	2.68E-15	0.012	1.000	− 2.28	5.46E−06	− 15.19	6.47E−11	13.70	4.12E−43	− 2.13	1.000	− 14.10	1.20E−15	− 9.84	8.28 E−09
*MKI67*	− 0.03	1.000	− 1.53	0.322	− 0.82	0.045	− 1.98	0.064	− 0.43	1.000	− 2.99	0.018	− 1.26	2.51E−09	− 1.71	0.021

Commonly modified genes in lidocaine-treated cancer cell lines from RNA-seq.

m_value, log2 fold change (with and without treatment); q_value, false discovery rate (FDR).

### Lidocaine increased expression and secretion of pro-protein GDF-15

**Figure 3:**
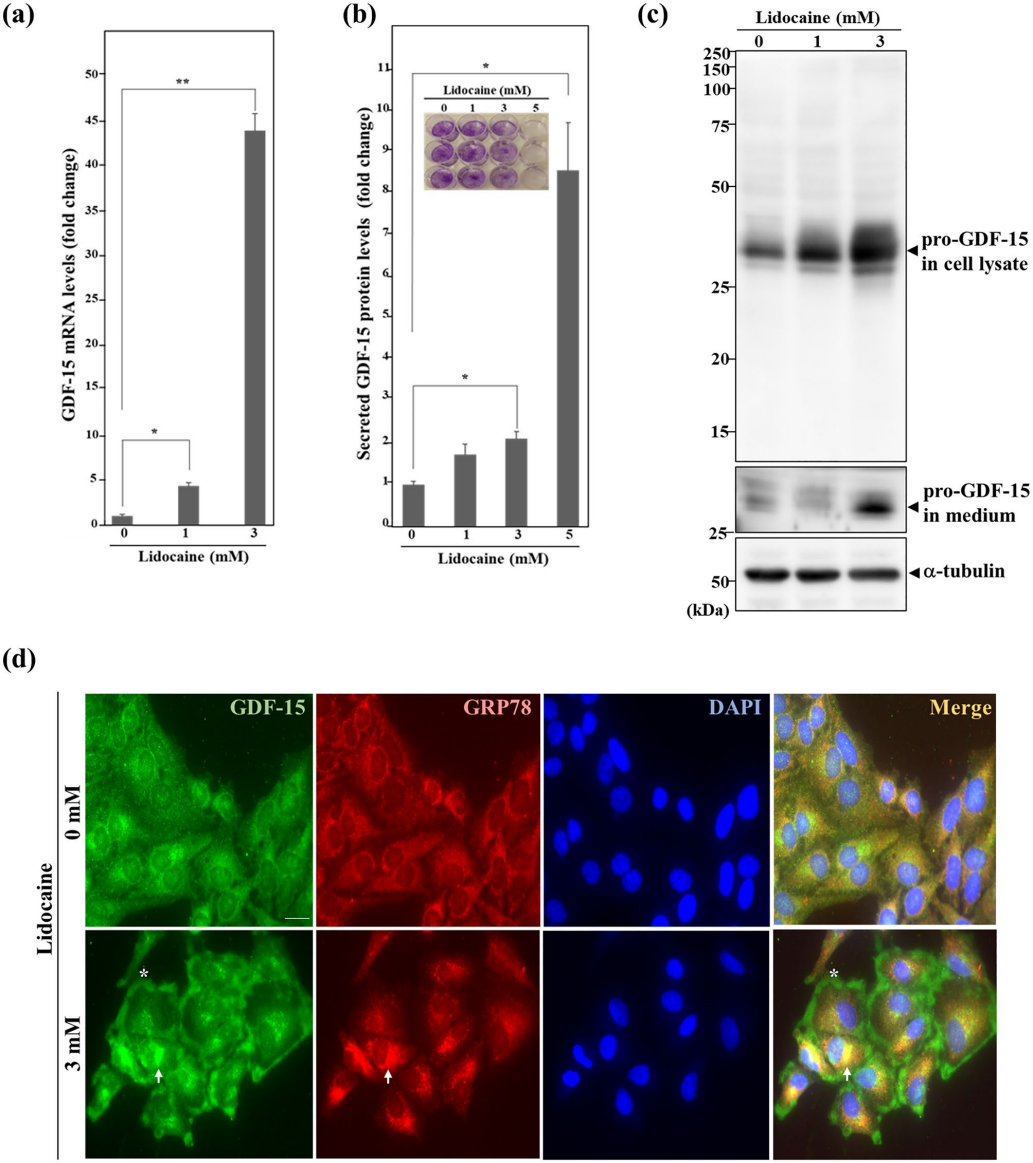
Increased expression and secretion of pro-protein GDF-15 with lidocaine treatment. (**a**) Level of GDF-15 mRNA after lidocaine treatment by qPCR. Total RNA purified from lidocaine-treated HeLa cells for 24 h were used for reverse transcription and qPCR analysis using GDF-15 and GAPDH primers. The results were normalized by the expression levels of GAPDH at the indicated lidocaine concentration. The level of GDF-15 mRNA increased with lidocaine treatment. Each sample was assayed in triplicate and the results are presented as the mean ± SD. *p < 0.05, **p < 0.001. (**b**) ELISA of GDF-15 using culture medium. HeLa cells were treated with the indicated concentration of lidocaine for 24 h. The results were normalized by the cell number, determined by staining the culture wells with 0.5% crystal violet. Secreted GDF-15 in the medium increased after lidocaine treatment. Each sample was assayed in triplicate. The results represent the mean ± SD. *p < 0.05. (**c**) Western blotting of GDF-15. Cell lysate and culture medium of HeLa cells treated with the indicated concentration of lidocaine for 24 h were subjected to blotting using GDF-15 and α-tubulin antibodies. Pro-protein form GDF-15 (pro-GDF-15, 34 kDa) in both cell lysate and medium increased after lidocaine treatment. (**d**) Immunofluorescence analysis using GDF-15 and GRP78 antibodies. GRP78 is an ER chaperon, serving as an ER marker. HeLa cells treated with 3 mM lidocaine for 24 h were used for assay. Nuclei were stained with DAPI. The arrow indicates increased GDF-15 in the ER, and the asterisk indicates GDF-15 accumulation in the pericellular region. The scale bar represents 10 μm.

To confirm the increased expression of GDF-15, qPCR was performed using lidocaine-treated HeLa cells. Lidocaine induced GDF-15 expression in a concentration-dependent manner and the GDF-15 level significantly increased by about 45-fold with 3 mM lidocaine treatment compared with that in untreated HeLa cells (Fig. 3a). We next examined the levels of secreted GDF-15 by enzyme-linked immunosorbent assay (ELISA) using the culture medium of lidocaine-treated HeLa cells. After normalization of GDF-15 levels by cell number, it was revealed that lidocaine increased the extracellular secretion of GDF-15. Treatment with 5 mM lidocaine almost completely suppressed the growth of HeLa cells, although the secretion of GDF-15 increased about eightfold, suggesting that lidocaine-induced growth inhibition correlates with increased secretion of GDF-15 (Fig. 3b). We further performed Western blotting using GDF-15 polyclonal antibodies purified from rabbits immunized with GDF-15 peptide (30–308 aa). The use of cell lysates and culture medium treated with lidocaine resulted in an increase of pro-protein GDF-15 (34 kDa) both intracellularly and in the culture medium (Fig. 3c). These results indicated that lidocaine-induced GDF-15 was at least pro-protein form and was secreted extracellularly in a concentration-dependent manner. In addition, we investigated the cellular distribution of lidocaine-induced GDF-15. Immunofluorescence analysis showed that GDF-15 was localized both in the nucleus and cytoplasm in lidocaine-untreated HeLa cells. After treatment with 3 mM lidocaine, increased GDF-15 was observed around the perinuclear region. Co-staining of the ER chaperon glucose-regulated protein 78 (GRP78) revealed that increased GDF-15 was localized in the ER. In addition, the expression level of GRP78 was increased in lidocaine-treated HeLa cells in immunofluorescence assay although its level of mRNA was not increased in RNA-seq analysis, indicating that GRP78 was upregulated at translational level after lidocaine treatment (Fig. 3d, Supplementary Table 1). Furthermore, increased GDF-15 accumulated in the pericellular region in lidocaine-treated HeLa cells (Fig. 3d). These results demonstrate that lidocaine induced the expression and secretion of pro-protein form of GDF-15.

### Lidocaine-induced growth inhibition is suppressed by knockdown of pro-protein GDF-15

**Figure 4:**
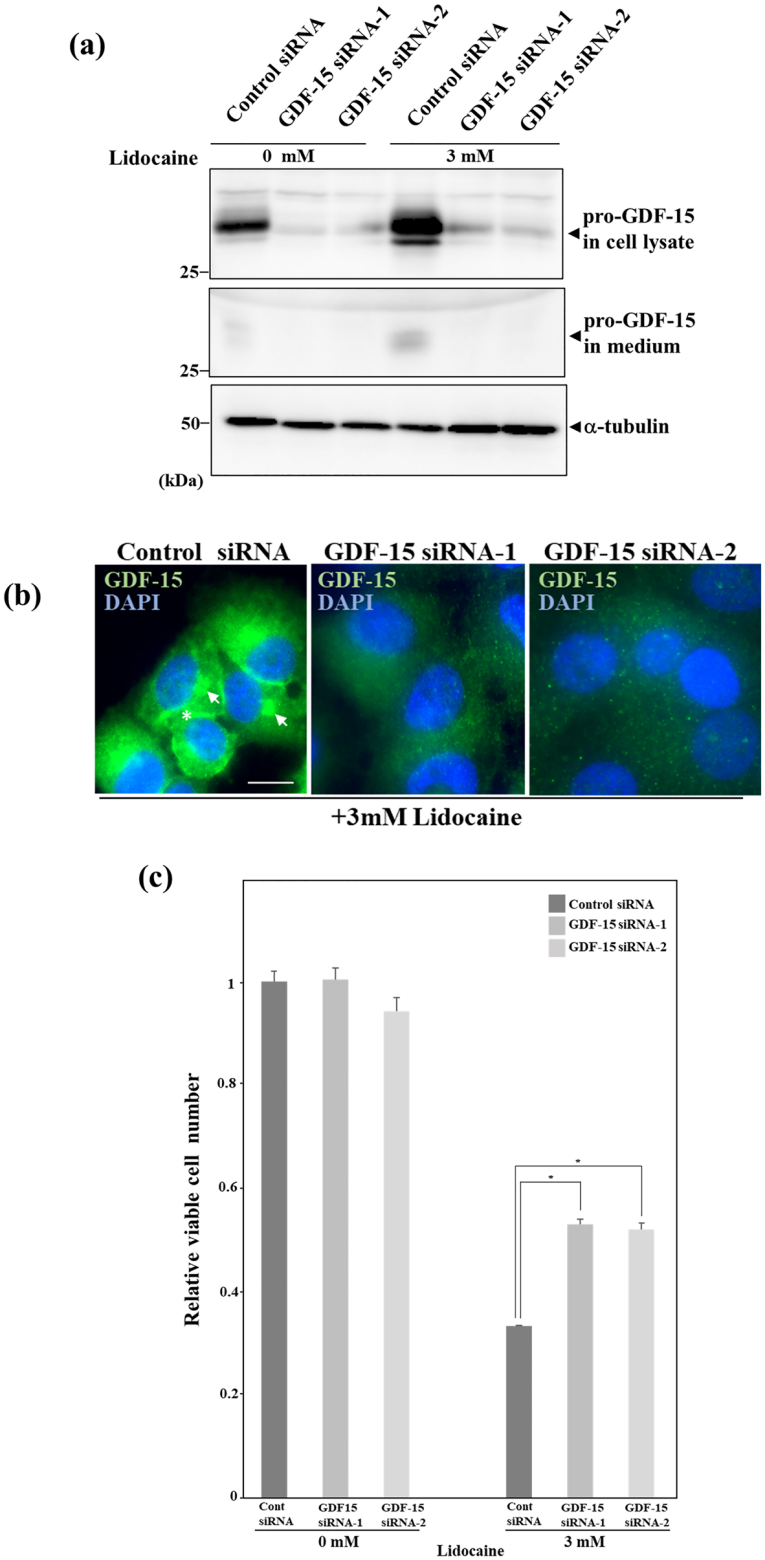
Knockdown of pro-protein GDF-15 suppresses lidocaine-induced growth inhibition. (**a**) Knockdown of GDF-15 by siRNA. Twenty-four hours after transfection with control or GDF-15 siRNA, HeLa cells were treated with the indicated dose of lidocaine for 24 h. Cell lysates and culture medium were applied for Western blotting using GDF-15 and α-tubulin antibodies. Pro-protein GDF-15 expression in cell lysates and culture medium was suppressed by GDF-15 siRNA1 and 2. (**b**) Immunofluorescence assay of GDF-15 knockdown of HeLa cells. Twenty-four hours after transfection with control or GDF-15 siRNA, HeLa cells were treated with 3 mM lidocaine for 24 h. After lidocaine treatment of HeLa cells transfected with control siRNA, lidocaine-induced GDF-15 was detected in the perinuclear (arrow) and pericellular region (asterisk) as shown in Fig. 3d (Fig. 5, left panel). After lidocaine treatment, GDF-15 expression was suppressed in HeLa cells transfected with GDF-15 siRNA 1 and 2 (middle and right panels). Nuclei were stained with DAPI. The scale bar represents 10 μm. (**c**) Cell growth in GDF-15 knockdown of HeLa cells after lidocaine treatment. Twenty-four hours after transfection with control or GDF-15 siRNA, HeLa cells were treated with the indicated concentration of lidocaine for 24 h. Cell growth was evaluated using 0.5% crystal violet staining. Lidocaine-induced growth inhibition was suppressed in GDF-15 siRNA transfected HeLa cells. Each sample was assayed in triplicate. The results are presented relative to the growth of lidocaine-untreated HeLa cells after transfection with control siRNA and expressed as the mean ± SD. *p < 0.05.

We examined the effect of GDF-15 on the growth of HeLa cells by knockdown of GDF-15 expression using two kinds of small interference RNA (siRNA). Lidocaine induced pro-protein GDF-15 expression and secretion in control siRNA-transfected HeLa cells. Conversely, its expression and secretion were suppressed in GDF-15 siRNA-transfected HeLa cells. These cells did not show a significant increase of GDF-15 after treatment with 3 mM lidocaine (Fig. 4a). Immunofluorescence assay showed that GDF-15 was observed in the perinuclear and pericellular regions in control HeLa cells after lidocaine treatment, as shown in Fig. 3d (Fig. 4b, left panel). In GDF-15 knockdown HeLa cells, GDF-15 was hardly detected after lidocaine treatment (Fig. 4b, middle and right panels). These results suggest that GDF-15 siRNA effectively suppressed lidocaine-induced pro-protein GDF-15 expression. We then investigated the growth of GDF-15 knockdown HeLa cells and lidocaine induced growth suppression in HeLa cells transfected with control and GDF-15 siRNA. However, lidocaine-induced growth inhibition was reduced by knockdown of pro-protein GDF-15, indicating that lidocaine-induced pro-protein GDF-15 causes growth suppression (Fig. 4c).

### Lidocaine induces apoptosis and TRIB3 expression

**Figure 5:**
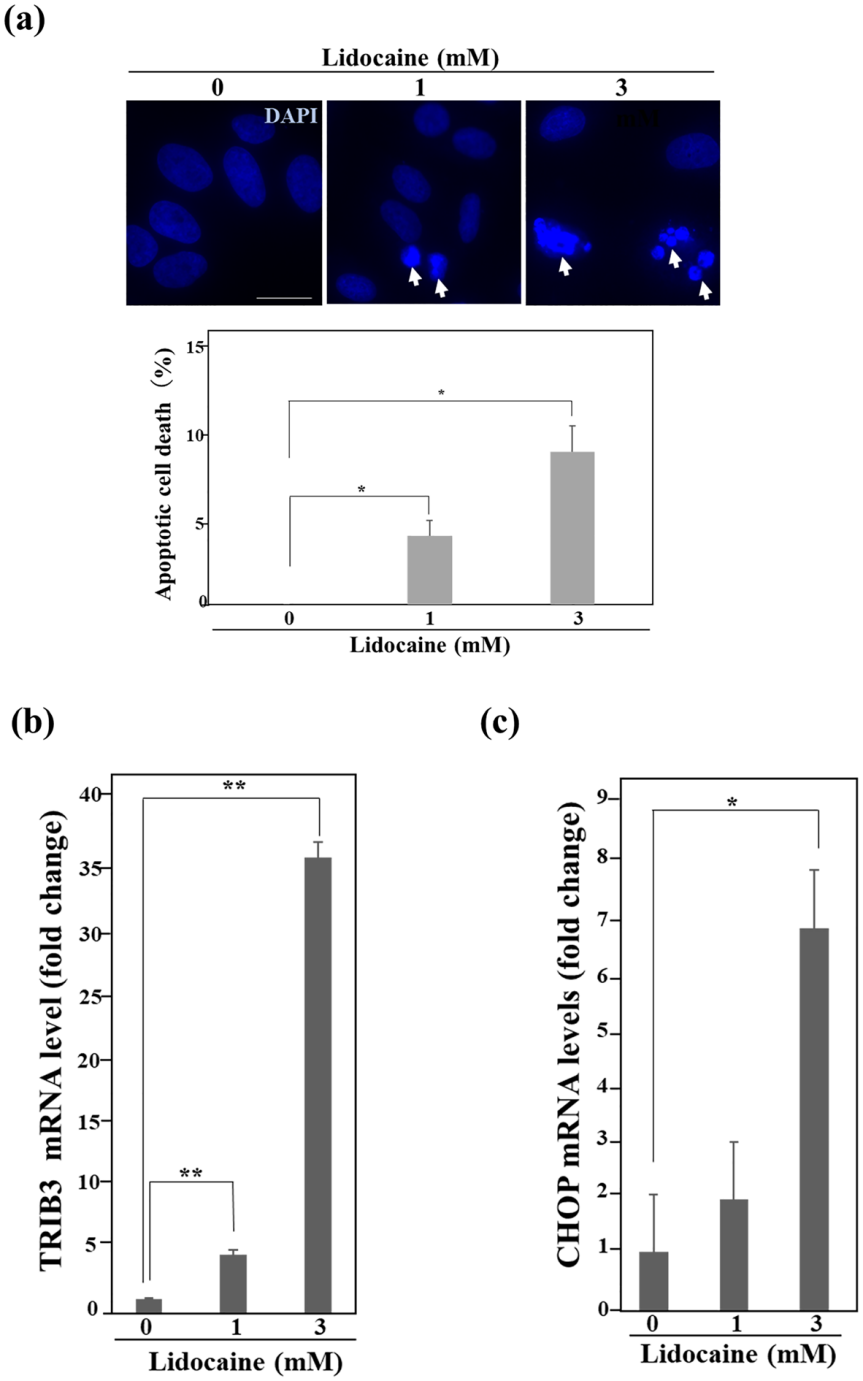
Lidocaine induces apoptosis and TRIB3 expression. (**a**) Number of apoptotic cells with chromosome condensation. Nuclei of HeLa cells treated with the indicated concentration of lidocaine for 24 h were stained with DAPI. The arrows indicate condensed chromosome. Each sample of one hundred cells was counted in triplicate. The number of apoptotic cells increased with lidocaine treatment in a concentration-dependent manner. The scale bar represents 10 μm. The results are presented relative to the total cell number and mean ± SD. *p < 0.05. (**b**) and (**c**) The level of TRIB3 and CHOP mRNA in qPCR. Total RNAs purified from HeLa cells treated with the indicated dose of lidocaine for 24 h were used for reverse transcription and qPCR analysis using TRIB3 or CHOP primers. The results were normalized by the expression levels of GAPDH at the indicated lidocaine concentration. The level of TRIB3 and CHOP mRNA increased with lidocaine treatment in a lidocaine-concentration dependent manner. Each sample was assayed in triplicate and the results are presented as the mean ± SD. *p < 0.05, **p < 0.001.

Since lidocaine caused growth suppression in cancer cell lines, we examined whether apoptosis was induced in lidocaine-treated HeLa cells. We observed that apoptotic cells with a condensed chromosome were hardly observed in lidocaine-untreated HeLa cells, while such cells increased in lidocaine-treated cells in a dose-dependent manner (Fig. 5a). RNA-seq revealed that apoptosis-inducible TRIB3 was also significantly upregulated as a commonly modified gene in the examined cancer cell lines with lidocaine treatment (Fig. 2b, Table 1). qPCR analysis showed that TRIB3 expression in HeLa cells treated with 3 mM lidocaine significantly increased by approximately 35-fold compared with that in untreated HeLa cells (Fig. 5b). In addition, transcription factor CHOP transactivates not only GDF-15 but also TRIB3 expression and lidocaine-treated HeLa and U2OS cells exhibited the tendency that CHOP expression was increased in a lidocaine-concentration dependent manner (Supplementary Table 1)^27,32^. qPCR showed that CHOP expression was increased in a lidocaine-concentration dependent manner and its expression in HeLa cells treated with 3 mM lidocaine was upregulated by approximately sevenfold compared with that in untreated HeLa cells, indicating that CHOP is involved in lidocaine-induced gene expression in HeLa cells (Fig. 5c).

### Lidocaine-induced apoptosis is suppressed by TRIB3 knockdown

**Figure 6:**
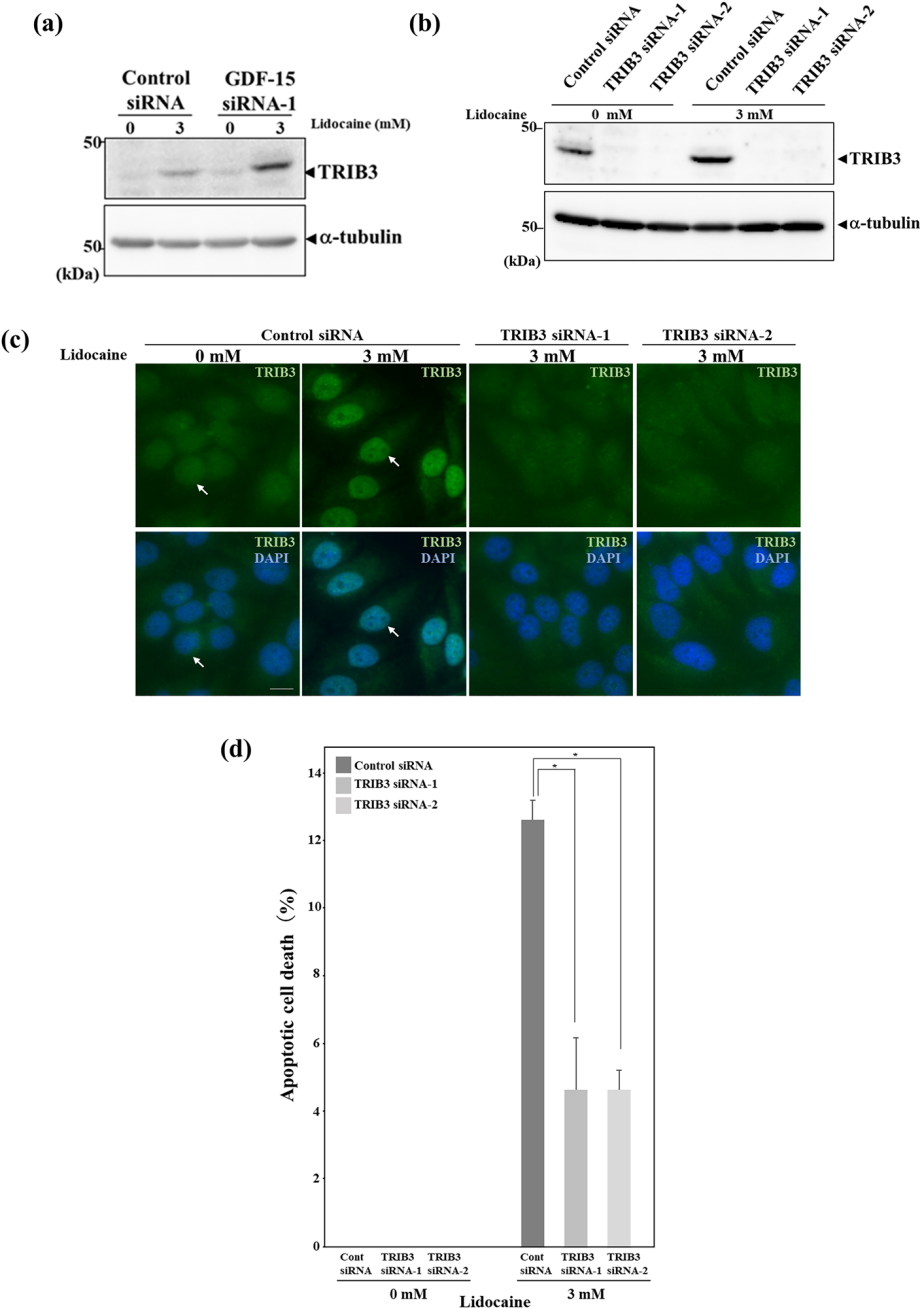
Knockdown of TRIB3 suppresses lidocaine-induced apoptosis. (**a**) TRIB3 expression increased in GDF-15 knockdown HeLa cells after lidocaine treatment. Twenty-four hours after transfection with control or GDF-15 siRNA, HeLa cells were treated with the indicated dose of lidocaine for 24 h. Cell lysates were used for Western blotting using TRIB3 and α-tubulin antibodies. TRIB3 expression increased with lidocaine treatment not only in control but also GDF-15 in siRNA-1 transfected HeLa cells. (**b**) Knockdown of TRIB3 expression by siRNA in Western blotting. Twenty-four hours after transfection with control or TRIB3 siRNA, HeLa cells were treated with the indicated dose of lidocaine for 24 h. Cell lysates were used for Western blotting using TRIB3 and α-tubulin antibodies. TRIB3 was detected in control siRNA-transfected HeLa cells and its expression increased after 3 mM lidocaine treatment. TRIB3 expression was suppressed by TRIB3 siRNA 1 and 2 with and without lidocaine treatment. (**c**) Knockdown of TRIB3 expression by siRNA in immunofluorescence assay. Twenty-four hours after transfection with control or TRIB3 siRNA, HeLa cells were treated with the indicated dose of lidocaine for 24 h and used for immunofluorescence assay using TRIB3 antibody. TRIB3 was detected in the nucleus (arrow in the left panel) and its expression increased after lidocaine treatment (arrow in the second panel from the left). TRIB3 expression was suppressed by TRIB3 siRNA 1 and 2 after lidocaine treatment (right two panels). Nuclei were stained with DAPI. The scale bar represents 10 μm. (**d**) Apoptosis decreased in TRIB3 knockdown HeLa cells after lidocaine treatment. Twenty-four hours after transfection with control or TRIB3 siRNA, HeLa cells were treated with the indicated dose of lidocaine for 24 h and used for assay. The number of apoptotic cells with chromosome condensation visualized by DAPI staining were counted. Each sample of one hundred cells was counted in triplicate. The results are presented relative to the total cell number and expressed as the mean ± SD. *p < 0.05.

**Figure 7:**
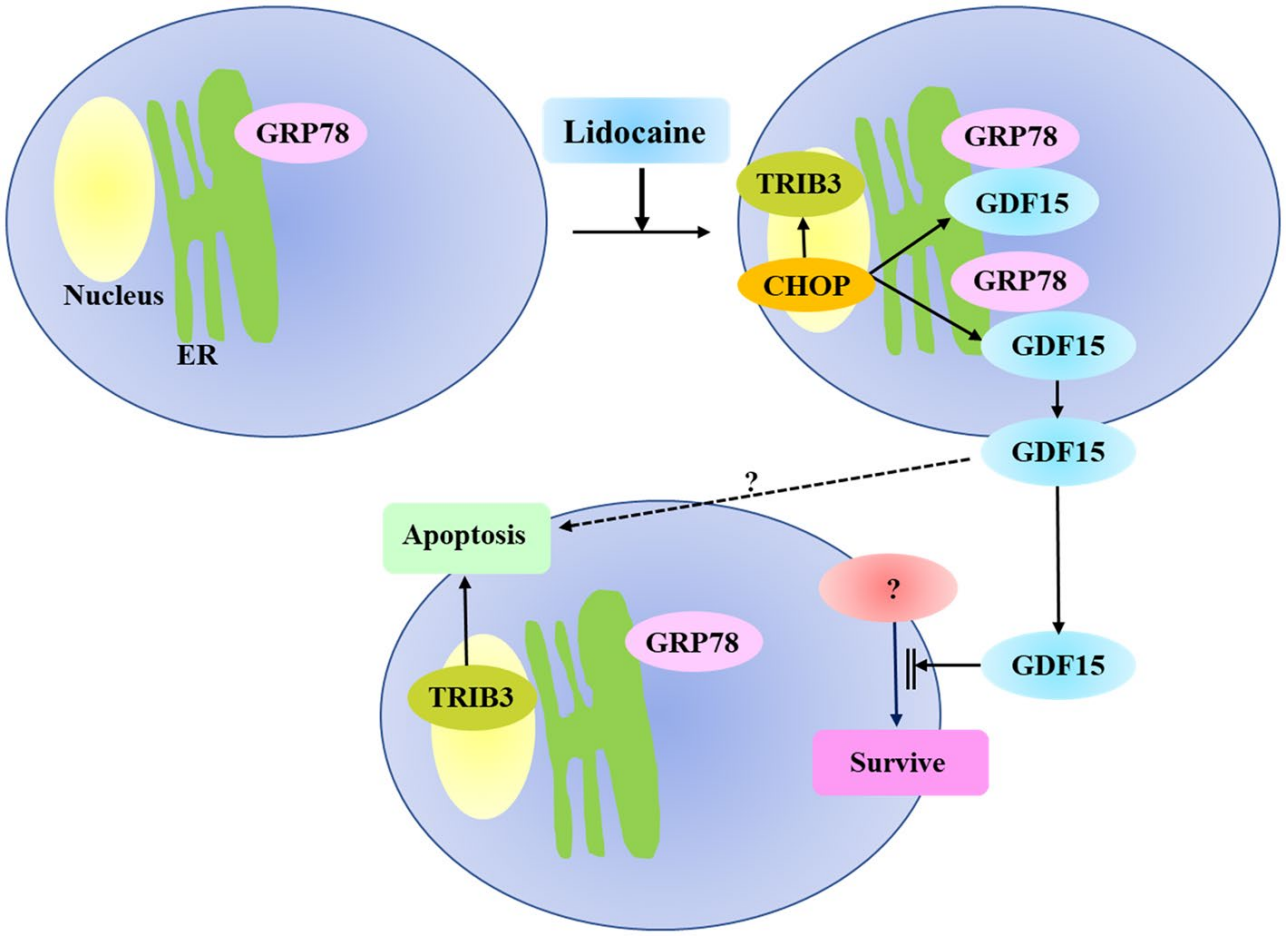
Proposed model of growth suppression and apoptosis caused by lidocaine-induced GDF-15 and TRIB3. Lidocaine induces expression of transcription factor CHOP, which transactivates GDF-15 and TRIB3. Lidocaine induced production of pro-protein form of GDF-15 (pro-GDF-15) in the ER and pro-GDF-15 that accumulates in the pericellular region is then secreted extracellularly. Secreted pro-GDF-15 might inhibit the survival signals or transmit apoptotic signals. Lidocaine also induces TRIB3 expression, which has a role in apoptosis.

Although lidocaine-induced growth suppression was effectively inhibited by GDF-15 knockdown, growth suppression was not completely inhibited. Thus, we examined the expression of TRIB3 in GDF-15 knockdown HeLa cells. TRIB3 expression increased with lidocaine treatment in both control and GDF-15 siRNA-transfected HeLa cells (Fig. 6a). To examine whether lidocaine-induced TRIB3 plays a role in the induction of apoptosis, we performed TRIB3 knockdown experiment. Western blotting using HeLa cells transfected with control siRNA showed an increase in TRIB3 expression after lidocaine treatment. On the other hand, TRIB3 expression was suppressed in HeLa cells transfected with TRIB3 siRNA with and without lidocaine treatment (Fig. 6b). We next examined TRIB3 expression by immunofluorescence assay. TRIB3 was expressed in the nucleus and its expression increased after lidocaine treatment in HeLa cells transfected with control siRNA, while its expression in the nucleus was suppressed in lidocaine-treated HeLa cells transfected with TRIB3 siRNA (Fig. 6c). Apoptotic cells were also increased in control siRNA transfected HeLa cells after lidocaine treatment. However, the number of lidocaine-induced apoptotic cells decreased in HeLa cells in which TRIB3 expression was suppressed by siRNA compared with HeLa cells transfected with control siRNA, indicating that lidocaine-induced TRIB3 plays a role in the induction of apoptosis (Fig. 6d). Taken together, lidocaine-induced pro-protein form of GDF-15 plays a role in growth suppression in HeLa cells, which accompanied with expression of apoptosis-inducible TRIB3 (Fig. 7).

## Discussion

In this study, we demonstrated that lidocaine induces growth suppression of cancer cell lines through increasing of GDF-15 expression. Additionally, lidocaine-induced GDF-15 was revealed to be pro-protein form of GDF-15 (pro-GDF-15). This form is known to be more rapidly secreted than the mature form^36^. Normal thyroid cells express pro-GDF-15, whereas papillary thyroid tumors express mature GDF-15 in large amounts^37^. Furthermore, decrease of pro-GDF-15 stored in the stroma of prostate cancer cells leads to poor outcomes after prostatectomy^15^. In addition, mutation of the pro-peptide region of GDF-15 (N-terminal region of pro-GDF-15) is involved in the poor prognosis of oral squamous cell carcinoma^38^. In our study, the GDF-15 knockdown experiment using siRNAs revealed that decreased pro-GDF-15 suppressed lidocaine-induced growth inhibition of HeLa cells, indicating that lidocaine-induced pro-GDF-15 might play a significant role in tumor suppression.

Although GDF-15 has tumor suppressive effects, increased serum GDF-15 in cancer patients indicates advanced disease^39^. A study of the effect of GDF-15 through glial cell-derived neurotrophic factor (GDNF) family receptor α-like protein (GFRAL) demonstrated that increased GDF-15 is related to the anorexia/cachexia observed in advanced cancer patients^40^. On the other hand, the GDF-15 receptor related to tumor suppression is still unidentified. In cervical cancer cell lines, including HeLa cells, GDF-15 promotes proliferation through phosphorylation of ErbB2, a member of the epithelial growth factor (EGF) receptor, and activation of PI3K/Akt and ERK signaling pathways^41^. Although this appears to be inconsistent with the results of our study, we speculate that the receptor for lidocaine-induced pro-GDF-15 might be different from ErbB2. A study using purified membrane fractions reported that a small fraction of GRP78 exists on the cell surface (CS-GRP78) transmitting Akt-mediated survival signaling and another study reported that monoclonal antibodies against for CS-GRP78 inhibit this survival signaling^42,43^. Lidocaine-induced pro-GDF-15 might have the potential to modify CS-GRP78-Akt-mediated survive signal. In addition, increased pro-GDF-15 in the ER might prevent GRP78 translocation from the ER to the cell surface.

In cancer cells, unregulated protein production disrupts fundamental ER functions such as protein folding, resulting in a state known as ER stress. Previous study reports that lidocaine causes ER stress-related apoptosis in adrenal pheochromocytoma PC12 cells^44^. Other amide-linked local anesthetics, such as bupivacaine and levobupivacaine inhibit the growth of colon cancer Caco-2 cells with activation of the ER stress response^45^. In addition, lidocaine upregulated CHOP, which is ER stress-related transcription factor induced by activating transcription factor 4 (ATF4) following ER stress sensor PKR-like ER kinase (PERK) activation^31^. Thereby, we examined whether lidocaine induces ER stress in HeLa cells. ER stress sensors such as PERK, inositol-requiring enzyme 1 (IRE1) and activating transcription factor 6 (ATF6) are activated by autophosphorylation or cleavage in ER stress^31^. Although phosphorylation of IRE1 was slightly increased, phosphorylation of PERK was hardly observed in lidocaine-treated HeLa cells (Supplementary Fig. 4a and b). In addition, ATF6 cleavage was not significantly increased though ATF6 expression was slightly increased in these cells (Supplementary Fig. 4c). Actually, only a few of ER stress-related molecules was upregulated in RNA-seq using lidocaine-treated cancer cell lines (Supplementary Table 1). Since ER stress is induced with treatment of 10 mM lidocaine in PC12 cells, treatment of such high concentration of lidocaine might be necessary to induce ER stress in cancer cell lines including HeLa cells. On the other hand, Tsai et al. reported that ER stress-inducible isochaihulactone, a natural compound extracted from herb induces apoptosis through induction of DDIT3 (CHOP), which is independent of GRP78 and PERK expression in glioblastoma multiform cells, indicating the existence of CHOP-mediated apoptosis pathway which is independent of the canonical ER stress signaling^46^. Further experiments are necessary to verify lidocaine-inducible ER stress in cancer cells.

Capsaicin-induced TRIB3 expression causes apoptosis through preventing AKT activity by inhibition of its phosphorylation^47^. In addition, capsaicin-induced GDF-15 causes apoptosis in colon cancer cell lines such as HCT116 cells^48^. These reports suggest that not only GDF-15 and but also TRIB3 is important in regulating the proliferation of cancer. Although, we could not elucidate the precise correlation between GDF-15 and TRIB3 in growth inhibition or apoptosis of HeLa cells, further experiments including other commonly modified genes should be performed to explain the roles of lidocaine in cancer pathology (Fig. 2b, Table 1)^49^.

In conclusion, our study indicates the possibility that locally administered lidocaine for pain control in cancer patients might suppress tumor growth by increasing GDF-15 and TRIB3 expression.

## Methods

### Cell cultures and reagents

Human cervical cancer HeLa (RCB0007) and hepatocellular carcinoma HepG2 (RBC1648) cells were provided by the RIKEN BioResearch Resource Center (BRC) through the National Bio-Resource Project of MEXT, Japan. Human osteosarcoma U2OS and colon cancer SW480 cells were purchased from American Type Culture Collection (VA, USA). HeLa, HepG2 and U2OS cells were cultured in Dulbecco Modified Eagle medium and SW480 cells were cultured in Leibovitz’s L-15 medium (Fujifilm-Wako, Osaka, Japan) supplemented with 10% (v/v) fetal bovine serum (Atlas Biologicals, CO, USA) at 37 °C under 5% CO_2_. Lidocaine hydrochloride isotonic solution (2%w/v) (Aspen, Tokyo, Japan) was added to the culture medium to achieve the test concentrations. PBS was used to adjust the total dose of lidocaine in each sample.

### Colony formation assay

Cells cultured in a six-well dish at the concentration of 3 × 10^4^ cells per well for two days were treated with lidocaine and cultured for a further 7 days. Cells fixed with 4% paraformaldehyde were stained with 0.5% crystal violet. For quantification of the colony number, the stained cultures dishes were treated with 10% acetic acid and absorbance of the solution was measured at 592 nm.

### Transcriptome expression analysis (RNA-seq)

Each cell line was cultured in a 60 mm culture dish at the concentration of 8 × 10^5^ cells per dish for 24 h and was treated with different concentrations of lidocaine. HeLa and U2OS cells were treated with 1 and 3 mM lidocaine, SW480 cells were treated with 1 and 5 mM lidocaine, and HepG2 were treated with 0.5 and 1 mM lidocaine. After treatment with lidocaine for 24 h, total RNAs from the cultured cells were extracted using a FastGene RNA Premium kit (Nippon Genetics Inc., Tokyo, Japan) according to the manufacturer’s protocol. Purified RNA quality was evaluated by the RNA integrity number (RIN) using Agilent RNA6000 Pico Kit and the Agilent 2100 Bioanalyzer (Agilent Technologies, Santa Clara, CA, USA). High-quality RNA samples (RIN > 7.0) were used for RNA-seq analysis. One microgram of total RNA was applied for preparing sequence libraries using a KAPA mRNA HyperPrep Kit (Kapa Biosystems Inc., Wilmington, MA, USA) following the manufacturer’s instructions. The generated libraries and 1% PhiX spike-in were then subjected to paired-end sequencing of 43-bp reads using a NextSeq500 System (Illumina Inc., San Diego, CA, USA) with a NextSeq500 High Output v2.5 Kit (Illumina). The reads were aligned to the UCSC human reference genome 19 (hg19) for calculation of expression values (read counting and TPM (Transcripts Per Million) normalized value) using the STAR (version 2.5.3a)-RSEM (version 1.3.3) pipeline with the following options: -paired-end, -star, -star-gzipped-read-file, -strandedness reverse, -output-genome-bam, -sort-bam-by-coordinate, and -estimate-rspd. The average values of the number of input reads and number of uniquely mapped reads were approximately 35.1 M (range 33.3–37.2 M) and 29.6 M (range 28.1–31.3 M), respectively. Differentially expressed genes (DEGs) between two conditions were detected using TCC-iDEGES-DESeq pipeline on R (https://www.R-project.org/, version 4.0.3) package TCC (version 1.26.0), and pathway analysis was conducted using Ingenuity Pathway Analysis (IPA, QIAGEN, Germantown, MD, USA). Genes with a false discovery rate (FDR, adjusted *p* value) of < 0.05 were defined as being significant DEGs. Heatmaps were generated based on log2 fold change values with an adjusted *p* value of < 0.05 in at least one analysis, which was obtained from TCC-iDEGES-DESeq using the heatmap2 library on R package gplots_3.1.1 as follows: calculating z-scores, omitting “N/A” data, and hierarchical clustering with Ward’s method. Sequence data are available through ArrayExpress under the accession number E-MTAB-11320.

### Quantitative PCR (qPCR)

Reverse transcription of total RNA purified from HeLa cells was performed using PrimeScript (Takara Bio Inc., Osaka, Japan) following the manufacturer’s protocol. After incubation at 50 °C for 2 min and 95 °C for 2 min, PCR cycles were performed at 95 °C for 15 s, 60 °C for 15 s and 72 °C for 1 min using PowerUp SYBR Green Master Mix on a StepOne Real Time PCR system (ThermoFisher Scientific, MA, USA). The sequences of primers used for PCR were as follows: forward of GDF-15 was 5’-CAGTCGGACCAACTGCTGGCA-3’ and reverse of GDF-15 was 5'-TGAGCACCATGGGATTGTAGC-3', forward of CHOP was 5'-TCTAAGGCACTGAGCGTATCATGT-3' and reverse of CHOP was 5'-TTTCAGGTGTGGTGATGTATGAAG-3', forward of TRIB3 was 5'-GATCTCAAGCTGTGTCGCTTTGTC-3', and reverse of TRIB3 was 5'-GGGAATCATCTGGCCCAGTC-3', forward of GAPDH was 5'-AGGCTAGCTGGCCCGATTTC-3' and reverse of GAPDH was 5'-TGGCAACAATATCCACTTTACCAGA-3'.

### ELISA

HeLa cells were cultured in a twelve-well dish at the concentration of 4 × 10^4^ per well for two days. Cells were treated with lidocaine at the indicated concentration for 24 h and the culture medium was applied to the human GDF-15 immunoassay kit (Proteintech, Rosemont, IL, USA) following the manufacturer’s instructions. Samples were incubated with anti-GDF-15 antibodies coated on a microplate for 2 h at 37 °C, following which the wells were washed four times. Next, the wells were incubated with GDF-15 antibody conjugated with horseradish peroxidase for 1 h at room temperature. After washing, the wells were incubated with 3,3',5,5'-tetramethyl-benzidene (TMB) substrate solution for 30 min at room temperature. After addition of the sulfuric acid stop solution, the intensity of the solution in each well was measured at 450 nm.

### Antibodies

Antibodies used in this study were rabbit anti-GDF-15 antibody (Proteintech, IL, USA), mouse anti-GRP78 and mouse anti-α-tubulin antibody (DM1A) (SantaCruz Biotech, TX, USA), rabbit anti-TRIB3 antibody (ab137526, Abcam), goat Cy3-conjugated anti-rabbit IgG antibody, and donkey Cy2-conjugated anti-mouse IgG antibody (Jackson ImmunoResearch Laboratories, PA, USA).

### Western blotting

HeLa cells were lysed with RIPA buffer (50 mM Tris–HCl, pH 8.0, 150 mM NaCl, 0.1% SDS, 0.5% deoxycholate and 1% Triton X-100) supplemented with 2 mM PMSF, protease inhibitor cocktail and 1 mM sodium orthovanadate (SantaCruz Biotech, TX, USA). The lysate was subjected to SDS-PAGE and protein was transferred to a polyvinylidene difluoride membrane (IPVH00010, Merck, Darmstadt, Germany). The membranes were blocked with 3% non-fat dry milk dissolved in Tris-buffered saline containing 0.05% Tween 20 (TBS-T). The membranes were cut prior to hybridization with primary antibodies (Figs. 3c, 6b and Supplementary Fig. 4a–c). A primary antibody reaction using Can Get Signal Immunoreaction Enhancer Solution (NKB101, Toyobo Biotechnology Co. Ltd., Osaka, Japan) was performed for 1 h at room temperature, and the membranes were washed with TBS-T. An HRP-conjugated secondary antibody reaction was performed at room temperature for 1 h. After washing the membranes, Western Lighting Plus (Perkin Elmer, MA, USA) and Merstham Imager 600 (Cytiva, Tokyo, Japan) were used for protein detection.

### Immunofluorescence assay

HeLa cells were cultured on cover glasses. The cells were fixed with 4% paraformaldehyde for 30 min and permeabilized with 0.2% Triton-X for 5 min at room temperature. 5% bovine serum albumin (BSA) was used for blocking for 60 min at room temperature. The primary antibody was incubated for 60 min at room temperature and washed three times with PBS. Fluorescence conjugated secondary antibody was incubated for 60 min at room temperature and washed three times with PBS. DNA was stained with 300 nM 4',6-diamidino-2-phenylindole (DAPI) (Invitrogen, MA, USA) for 15 min at room temperature. The images were observed by immunofluorescence microscopy (Nikon Eclipse Ni-E, Tokyo, JAPAN) equipped with NIS-Elements Ver 5.1 software.

### RNA interference

HeLa cells were cultured in a six-well plate at the concentration of 5 × 10^5^ per well for 24 h and transfected with siRNA using Lipofectamine RNAiMAX (ThermoFisher Scientific, MA, USA) following the manufacturer’s instructions. After 24 h from transfection, cells were treated with the indicated dose of lidocaine for 24 h and were applied for assay. Human GDF-15 siRNA-1 and siRNA-2 was designed as 5'-AUCCCAUGGUGCUCAUUCAdTdT-3' (position 871) and 5'-CCCGAUUCCUGCCCAAACAdTdT-3' (position 1049). Human TRIB3 siRNA-1 and siRNA-2 were designed as 5'-CCUGCAAGGUGUACCCCGUdTdT-3' (position 349) and 5'-CCCGAUCCCAUCUCUGGGAdTdT-3' (position 1039). Each siRNA sense and antisense strand was synthesized and annealed by NipponGene Inc. (Toyama, Japan). Control siRNA was purchased from NipponGene Inc.

### Statistical analysis

Values are presented as the mean ± standard deviation (SD). Significant differences among experimental samples were assessed using Bonferroni’s method. *p* values < 0.05 were considered to indicate a statistically significant difference.

## Supplementary Information

Supplementary Information 1.

Supplementary Information 2.

Supplementary Information 3.

Supplementary Information 4.

Supplementary Information 5.

Supplementary Information 6.

Supplementary Information 7.

Supplementary Information 8.

Supplementary Information 9.

Supplementary Information 10.

Supplementary Information 11.

Supplementary Information 12.

Supplementary Information 13.

Supplementary Information 14.

## Data Availability

RNA sequence data are available from ArrayExpress under the accession number E-MTAB-11320. https://www.ebi.ac.uk/arrayexpress/experiments/E-MTAB-11320/
