# Supplementary material for: Local anesthetic lidocaine-inducible gene, growth differentiation factor-15 suppresses the growth of cancer cell lines
Source: Sci Rep. 2022 Aug 25;12:14520. doi: 10.1038/s41598-022-18572-3 (PMC9411556; doi:10.1038/s41598-022-18572-3)
Supplement: Supplementary file 13 — Supplementary Information 13. [file 41598_2022_18572_MOESM13_ESM.docx]

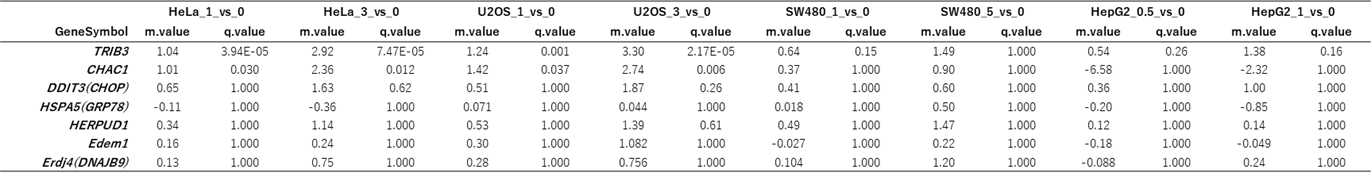


Altered gene expression associated with ER stress with exposure of cells to lidocaine from RNA-seq.

m_value, log2 fold change (with and without treatment); q_value, false discovery rate (FDR)

**Supplementary Table 1 Altered gene expression associated with ER stress with exposure of cells to lidocaine**
