# Supplementary material for: Local anesthetic lidocaine-inducible gene, growth differentiation factor-15 suppresses the growth of cancer cell lines
Source: Sci Rep. 2022 Aug 25;12:14520. doi: 10.1038/s41598-022-18572-3 (PMC9411556; doi:10.1038/s41598-022-18572-3)
Supplement: Supplementary file 14 — Supplementary Information 14. [file 41598_2022_18572_MOESM14_ESM.docx]

**Supplementary Figure Legends**

**Supplementary Figure 1a**

Uncropped images of Western blotting of GDF-15 (left panel) and α-tubulin (right lower panel) using the cell lysate in Figure 3c. In the GDF-15 blot, the membrane was cut prior to hybridization with anti-GDF-15 antibodies. The blot of GDF-15 shows membrane edges. In the α-tubulin blot, the membrane was cut into a piece containing molecular weight 34~60 kDa prior to hybridization with anti-α-tubulin antibodies, as can be seen in the bright field image of the membrane (right upper panel). The blotting area of α-tubulin surrounded by the dotted line box was used in Figure 3c. The blot of α-tubulin shows membrane edges though the left side of the blot was out of the frame when we took the image using a Merstham Imager. The arrowheads indicate pro-protein GDF-15 (left) and α-tubulin (right), respectively.

**Supplementary Figure 1b**

Uncropped images of Western blotting of GDF-15 using culture medium in Figure 3c. The blot of GDF-15 shows membrane edges. The blotting area within the dotted line box was used in Figure 3c. The arrowhead indicates pro-protein GDF-15 in culture medium.

**Supplementary Figure 2a**

Uncropped images of Western blotting of GDF-15 using cell lysate in Figure 4a. The blot of GDF-15 shows membrane edges. The arrowhead indicates pro-protein GDF-15 in cell lysates.

**Supplementary Figure 2b**

Uncropped images of Western blotting of GDF-15 using culture medium in Figure 4a. The blot of GDF-15 shows membrane edges. The arrowhead indicates pro-protein GDF-15 in culture medium.

**Supplementary Figure 2c**

Uncropped images of Western blotting of α-tubulin in Figure 4a. The blotting area surrounded by the dotted line box was used in Figure 4a. An enhanced image of the blot (right) shows membrane edges. The arrowhead indicates α-tubulin.

**Supplementary Figure 3a**

Uncropped images of Western blotting of TRIB3 in Figure 6a. The blot of TRIB3 shows membrane edges. The blotting area within the dotted line box was used in Figure 6a. The arrowhead indicates TRIB3.

**Supplementary Figure 3b**

Uncropped images of Western blotting of α-tubulin in Figure 6a. The blotting area within the dotted line box was used in Figure 6a. An enhanced image of the blot (right) shows membrane edges. The arrowhead indicates α-tubulin.

**Supplementary Figure 3c**

Uncropped images of Western blotting of TRIB3 in Figure 6b. The membrane was cut prior to hybridization with anti-TRIB3 antibodies. The blot of TRIB3 shows membrane edges. The arrowhead indicates TRIB3.

**Supplementary Figure 3d**

Uncropped images of Western blotting of α-tubulin in Figure 6b. The membrane was cut prior to hybridization with α-tubulin antibodies. An enhanced image of the blot (right) shows membrane edges. The arrowhead indicates α-tubulin.

**Supplementary Figure 4a**

Uncropped images of Western blotting of phosphorylated IRE1 and IRE1 using rabbit anti-phosphorylated IRE1 (Ser724) antibody (ab48187, Abcam, Cambridge, UK) and rabbit IRE1 antibody (ab37073, Abcam, Cambridge, UK). In the phosphorylated IRE1 and IRE1 blots, the membrane was cut prior to hybridization with anti-phosphorylated IRE1 and IRE1 antibodies. The blots show membrane edges. The arrowheads indicate phosphorylated IRE1 (left) and IRE1 (right), respectively.

**Supplementary Figure 4b**

Uncropped images of Western blotting of phosphorylated PERK and PERK using rabbit anti-phosphorylated PERK (Thr980) antibody (16F8) (Cell signaling Technology, MA, USA) and rabbit PERK antibody (C33E10) (Cell signaling Technology, MA, USA). In the phosphorylated PERK and PERK blots, the membrane was cut prior to hybridization with anti-phosphorylated PERK and PERK antibodies. The blot of phosphorylated PERK shows membrane edges. An enhanced image of the blot (right panel of PERK) shows membrane edges. The arrowhead indicates PERK.

**Supplementary Figure 4c**

Uncropped images of Western blotting of ATF6 and α-tubulin using mouse anti-ATF6 antibody (66563-Ig, Proteintech, IL, USA) and α-tubulin antibody. In the ATF6 and α-tubulin blots, the membrane was cut prior to hybridization with ATF6 and α-tubulin antibodies. An enhanced images of the blots (right panel of ATF6 and right panel of α-tubulin, respectively) show membrane edges. The arrowheads indicate ATF6 (left) and α-tubulin (right), respectively.
